# Enterotype-stratified gut microbial signatures in MASLD and cirrhosis based on integrated microbiome data

**DOI:** 10.3389/fmicb.2025.1568672

**Published:** 2025-05-15

**Authors:** Heng Yuan, Junyu Zhou, Xuangao Wu, Shiwei Wang, Sunmin Park

**Affiliations:** ^1^Department of Physiology and Pathophysiology, Xi’an Jiaotong University School of Basic Medical Sciences, Xi'an, China; ^2^Department of Bioconvergence, Hoseo University, Asan, Republic of Korea; ^3^Institute of Advanced Clinical Medicine, Peking University, Beijing, China; ^4^Key Laboratory of Resources Biology and Biotechnology in Western China, Ministry of Education, Provincial Key Laboratory of Biotechnology of Shaanxi Province, The College of Life Sciences, Northwest University, Xi’an, China; ^5^Department of Food and Nutrition, Obesity/Diabetes Research Center, Hoseo University, Asan, Republic of Korea

**Keywords:** MASLD, cirrhosis, gut microbiota, machine-learning approach, enterotypes

## Abstract

**Introduction:**

Metabolic dysfunction-associated steatotic liver disease (MASLD) is a growing global health challenge, characterized by significant variability in progression and clinical outcomes. While the gut microbiome is increasingly recognized as a key factor in liver disease development, its role in disease progression and associated mechanisms remains unclear. This study systematically investigated the gut microbiota’s role in MASLD and liver cirrhosis progression, focusing on individual bacterial strains, microbial community dynamics, and functional characteristics across different enterotypes.

**Methods:**

Publicly available next-generation sequencing(NGS) datasets from healthy individuals and patients with MASLD and cirrhosis were analyzed. Enterotype classification was performed using principal component analysis, with advanced bioinformatics tools, including Linear Discriminant Analysis Effect Size (LEfSe), eXtreme Gradient Boosting (XGBoost), and Deep Cross-Fusion Networks for Genome-Scale Identification of Pathogens (DCiPatho), to identify differentially abundant microbes and potential pathogens. Microbial co-occurrence networks and functional predictions via PICRUSt2 revealed distinct patterns across enterotypes.

**Results and discussion:**

The Prevotella-dominated(ET-P) group exhibited a 33% higher cirrhosis rate than the Bacteroides-dominated(ET-B) group. Unique microbial signatures were identified: *Escherichia albertii* and *Veillonella nakazawae* were associated with cirrhosis in ET-B, while *Prevotella copri* was linked to MASLD. In ET-P, *Prevotella hominis* and *Clostridium saudiense* were significantly associated with cirrhosis. Functional analysis revealed reduced biosynthesis of fatty acids, proteins, and short-chain fatty acids (SCFAs), coupled with increased lipopolysaccharide(LPS) production and altered secondary bile acid metabolism in MASLD and cirrhosis patients. There were significant microbial and functional differences across enterotypes in MASLD and cirrhosis progression, providing critical insights for developing personalized microbiome-targeted interventions to mitigate liver disease progression.

## Introduction

Metabolic dysfunction-associated steatotic liver disease (MASLD) is a complex metabolic liver disorder characterized by excessive fat accumulation in the liver. A 2024 study estimates the global prevalence of MASLD at approximately 32.4%, with notable variation across regions and between sexes—39.7% in men and 25.6% in women (Le et al., 2025). Projections suggest that by 2040, the prevalence could surpass 55%, highlighting the increasing public health burden and the pressing need for further epidemiological research on MASLD (Le et al., 2025). Individuals with MASLD are more likely to be obese, with additional comorbidities such as type 2 diabetes (T2DM), metabolic syndrome (MetS) and associated cardiovascular risk factors. MASLD is the early stage of a progressive condition, wherein fat accumulates in the liver cells, but there is minimal inflammation or liver damage. Most patients with MASLD may not experience significant symptoms or progressive liver damage. Some patients may progress to metabolic dysfunction-associated steatohepatitis (MASH) which can further progress to cirrhosis or hepatocellular carcinoma (Shao et al., 2022). Cirrhosis represents the end stage of chronic liver disease in which advanced liver fibrosis is seen. This can occur due to several liver diseases, including hepatitis and chronic alcohol consumption. Treatment of cirrhosis involves addressing the underlying cause and attempting to reverse liver fibrosis. However, no clear consensus exists on the most effective treatment for cirrhosis.

Emerging evidence reveals a complex relationship between MASLD and gut microbiota, with patients frequently exhibiting intestinal dysbiosis (Gudan et al., 2023). Gut microorganisms could potentially serve as early diagnostic markers and therapeutic targets for MASLD, MASH, and cirrhosis. The gut-liver axis represents a sophisticated bidirectional communication system wherein gut microbiota critically modulate liver health and disease progression (Tilg et al., 2022). A multifaceted investigation is essential to comprehensively elucidate the role of the microbiome in MASLD, MASH, and cirrhosis. Distinct microbial community clusters, known as enterotypes, have been identified in the human gut (Frioux et al., 2023). These enterotypes are characterized by the dominance of specific bacterial genera, such as Prevotella or Bacteroides, and are associated with unique functional profiles (Di Pierro, 2021). This approach encompasses three primary investigative perspectives: (1) Analysis of individual bacterial strains to identify the specific microorganisms that trigger liver pathogenesis, (2) Examination of community-wide ecological dysbiosis to understand intricate microbial composition shifts, and (3) Functional analysis based on enterotypes to understand how microbial community structures influence metabolic processes, inflammation, and liver damage (Zhao et al., 2023).

The gut microbiota functions as a dynamic ecosystem fundamental to maintaining immune system homeostasis, preventing autoimmunity, and defending against pathogenic invasion (Pickard et al., 2017). The liver and large intestines share an intimate connection through multiple functional and vascular associations, including the biliary tract, portal vein, systemic circulation, autonomous nervous system, and gut hormones (Brandl et al., 2017). Recent studies have documented progressive changes in gut microbial composition that correlate directly with liver disease severity, with gut microbiota dysbiosis disruptions becoming increasingly pronounced as cirrhosis advances (Yuan et al., 2024a; Yuan et al., 2024b).

In 2011, Arumugam et al. (2011) introduced the concept of enterotypes, categorizing the gut microbiome into three major types: Bacteroides-dominated (ET-B), Prevotella-dominated (ET-P), and Ruminococcus-dominated (ET-R). These enterotypes demonstrate remarkable stability and show minimal association with demographic factors, BMI, or lifestyle variations, including dietary patterns (Wu et al., 2021; Ren et al., 2023). Previous research has established notable associations between enterotypes and various disease phenotypes (Wu and Park, 2022; Park et al., 2023b; Wu et al., 2024). ET-B correlates with reduced microbial diversity and is linked to MASH, colorectal cancer, immunosenescence, and low-grade chronic inflammation (Xu et al., 2021; Park et al., 2023b). Conversely, ET-P has been associated with rheumatoid arthritis, T2DM, MASLD, and acquired immunodeficiency syndrome (Yuan et al., 2024b). Despite these insights, the precise mechanisms by which the gut microbiota contribute to MASLD and liver cirrhosis progression remain incompletely understood.

We aimed to systematically investigate the mechanisms by which the gut microbiota contributes to the development and progression from health to MASLD and cirrhosis, utilizing the comprehensive approach mentioned above that integrates strain-level analysis, community-wide ecological assessment, and functional enterotype characterization. This research offers potential for developing innovative diagnostic biomarkers and targeted therapeutic strategies, with the ultimate goal of providing personalized interventions that could improve patient outcomes and potentially halt or reverse the progression of metabolic liver disease by modulating the gut microbiota.

## Methods

### Search strategy

The primary objective was to systematically compare the gut microbiome composition across three distinct groups: healthy individuals, patients with MASLD, and those with liver cirrhosis. The systematic search and data extraction were conducted according to the Preferred Reporting Items for Systematic Reviews and Meta-Analyses (PRISMA) guidelines.^1^ With this objective, a comprehensive literature search was conducted across multiple specialized databases, including those of the European Bioinformatics Institute (EMBL-EBI, https://www.ebi.ac.uk/), the National Center for Biotechnology Information (NCBI, https://www.ncbi.nlm.nih.gov/), and the Gut Microbiota Repository (GMrepo, https://gmrepo.humangut.info/). The data collection included studies published up to August 2024. The search keywords included “non-alcoholic fatty liver disease” (NAFLD), “liver cirrhosis,” “gut microbiota,” “intestinal flora,” and their related synonyms, including emerging terminologies such as “metabolic associated fatty liver disease” (MAFLD) and “metabolic dysfunction-associated steatotic liver disease” (MASLD). Both medical subject heading (MeSH) terms and free text were utilized to ensure comprehensive coverage.

### Inclusion and exclusion criteria

Studies that followed specific diagnostic guidelines were included for the analysis as follows: The studies on MASLD were based on the “Multisociety Delphi Consensus Statement on New Fatty Liver Disease Nomenclature” (El-Kassas and Alswat, 2025), while the cirrhosis-related research complied with the “Evidence-based Clinical Practice Guidelines for Liver Cirrhosis 2020” (Yoshiji et al., 2021). All the selected studies were accorded Institutional Review Board approval and informed consent was obtained from the participants. To maintain research integrity and minimize potential confounding factors, we implemented strict exclusion criteria. Studies were excluded if participants had co-morbid conditions such as viral hepatitis, hypertension, diabetes, or heart disease, or had taken antibiotics within the preceding 3 months. Furthermore, to ensure methodological consistency and comparability, we exclusively selected studies that utilized 16S rRNA gene amplicon sequencing as the primary investigative technique.

### Downloading data and annotating species

Raw 16S rRNA sequencing data were obtained from the Sequence Read Archive (SRA) and converted into paired-end FASTQ format using the SRA Toolkit (v2.5.2). All datasets underwent a unified preprocessing pipeline implemented in the Quantitative Insights into Microbial Ecology Version 2 (QIIME2) platform (version 2021.2) for comprehensive 16S rRNA gene analysis. Quality control steps included removal of sequences with ambiguous bases, minimum Phred quality score >30, and a read depth threshold >10,000 reads per sample. Amplicon sequence variants (ASVs) were generated using the Divisive Amplicon Denoising Algorithm (DADA2) plugin with denoising, chimera removal (consensus method), and truncation lengths set at 240 bp (forward) and 200 bp (reverse) to ensure optimal quality. Taxonomic classification of ASVs was performed using a pre-trained naïve Bayes classifier, specifically comparing them against the Greengenes 13_8 database, focusing on the V4 region with a 99% similarity threshold. Only ASVs with a minimum frequency of 10 across all samples were retained for downstream analysis to minimize the impact of sequencing artifacts.

### Heterogeneity management and batch effect consideration

To account for potential heterogeneity across cohorts, we summarized each dataset’s characteristics—including sequencing platform, geographic origin, sample processing protocols, and diagnostic criteria—in Table 1. All datasets were processed through the same bioinformatic pipeline to minimize technical variability. Although formal batch correction tools such as ComBat or MMUPHin were not applied, we employed several strategies to mitigate cross-cohort confounding: First, unified quality control and processing: All samples were subjected to consistent quality thresholds and denoising steps, reducing pipeline-specific biases. Second, subgroup and stratified analyses: We performed subgroup analyses based on disease state and dataset origin to assess reproducibility of microbial patterns. Third, biological integration via enterotyping: Enterotype clustering based on principal component analysis (PCA) was used to group samples by microbial composition rather than study origin, helping reduce artificial clustering driven by technical variation.

**Table 1:** The characteristics of each selected three-arm cohort from health and MASLD and cirrhosis.

Study(ID)	Health	NAFLD	CIR	Year	Instrument	Gender(M/F)	Age	City, Country
PRJNA246121	32	53	0	2015	Illumina HiSeq 2000	32/53	45.00 ± 10.17	Beijing, China
PRJNA382861	25	31	0	2017	Illumina MiSeq	22/34	37.00 ± 6.33	Shanghai, China
PRJNA541489	23	24	0	2019	Illumina MiSeq	33/14	64.04 ± 7.30	Beijing, China
PRJNA518731	0	36	0	2020	Illumina MiSeq	–	–	Auckland, New Zealand
PRJNA559052	0	86	0	2020	Illumina MiSeq	56/30	50.80 ± 12.60	Leuven, Belgium
PRJEB40538	0	91	0	2021	Illumina MiSeq	35/26	48.8 ± 9.36	Italy; Greece; Serbia
PRJNA860335	29	32	0	2022	–	–	–	Beijing, China
PRJNA431746	8	0	88	2018	Illumina MiSeq	–	–	Styria, Austria
PRJNA445763	20	0	36	2018	–	–	–	Harbin, China
PRJNA449353	5	0	5	2019	–	–	–	Nanjing, China
PRJNA471972	14	0	35	2020	Illumina HiSeq4000	11/38	64.74 ± 9.80	Rome, Italy
PRJNA748675	1	0	1	2022	–	–	–	Taoyuan, Taiwan
PRJNA967488	0	0	21	2023	Illumina NovaSeq	8/13	57.14 ± 8.99	Chengdu, China
PRJEB41867	100	0	0	2020	Illumina HiSeq	48/52	24.1 ± 12.65	Graz, Austria
PRJNA736583	20	0	0	2022	Illumina MiSeq	10/10	53.2 ± 8.34	Naples, Italy
Asians	135	140	63					
Europeans	142	213	123					
Total	277	353	186					

### Enterotype classification and diversity analysis

Bioinformatic and statistical analyses were performed in R (v4.1.3). Gut microbial community structures were classified into enterotypes using principal component analysis (PCA) implemented via the FactoMineR and Factoextra packages. The optimal number of clusters was determined based on an eigenvalue threshold (>1.5) and validated using clustering performance metrics including the Calinski–Harabasz index and silhouette scores. Additionally, PCA-based permutation tests (*p*-value < 0.01) were conducted to assess the statistical significance of the clustering patterns.

Microbial alpha diversity was calculated using the Chao1 and Simpson indices, reflecting species richness and community evenness. Group-wise differences in microbial composition (healthy, MASLD, and cirrhosis) were evaluated using partial least squares discriminant analysis (PLS-DA) via the metaX package (v2.0.0). All data visualizations were generated using ggplot2 (v3.3.3) to effectively illustrate microbial community structure and diversity.

### Identification of microbial biomarkers

To identify core gut microbial signatures associated with liver disease progression, we implemented a multi-tiered filtering and machine learning framework. Initially, operational taxonomic units (OTUs) with relative abundance below 0.1% were excluded to reduce background noise. Differentially abundant taxa across disease stages were screened using Linear Discriminant Analysis Effect Size (LEfSe), incorporating Kruskal–Wallis testing followed by linear discriminant analysis.

Subsequently, we employed the eXtreme Gradient Boosting (XGBoost) algorithm to build a predictive model for disease classification based on microbial features. To enhance model interpretability, we applied SHapley Additive exPlanations (SHAP) to calculate the average absolute SHAP values of each OTU at the species level, revealing their relative importance in the classification process. Importantly, 5-fold cross-validation was implemented during model training to assess performance stability and mitigate overfitting. However, due to the absence of an external dataset with harmonized metadata and sequencing protocols, independent cohort validation could not be conducted.

### Construction of microbial co-occurrence networks and functional annotation

Microbial co-occurrence network construction was performed using FastSpar (version 0.0.10), calculating Spearman correlation coefficients (Sparcc) between microbial taxa to identify statistically significant correlations. The resulting network was visualized using Gephi (version 0.9.5), providing a comprehensive view of microbial interactions. Functional potential prediction of the gut microbiome was conducted using Phylogenetic Investigation of Communities by Reconstruction of Unobserved States (PICRUSt2), which maps 16S rRNA gene sequences to reference genomes in the Kyoto Encyclopedia of Genes and Genomes (KEGG) database. Functional profiles were standardized using Z-scores, with heatmaps generated through the pheatmap package.

To explore the intricate relationships between microbial biomarkers and biological functions, we performed correlation analyses using the ggplot2 and linkET packages, complemented by Mantel testing to assess the correlations between matrices. Additionally, the deep cross-fusion networks for genome scale identification of pathogens (DCiPatho) method was employed to evaluate the potential pathogenic impact, by calculating the product of relative abundance and virulence score to quantify the influence of virulent microbes under varying conditions (Jiang G. et al., 2023).

### Statistical analysis

All statistical analyses were performed using the SPSS software (version 13.0, SPSS Inc., Armonk, NY, United States). Unless otherwise specified, the differences among groups were assessed by one-way analysis of variance (ANOVA) followed by multiple comparisons among the liver disease states. Statistical significance was set at *p* < 0.05 after the Bonferroni correction.

## Results

### Screening and included studies

**Figure 1:**
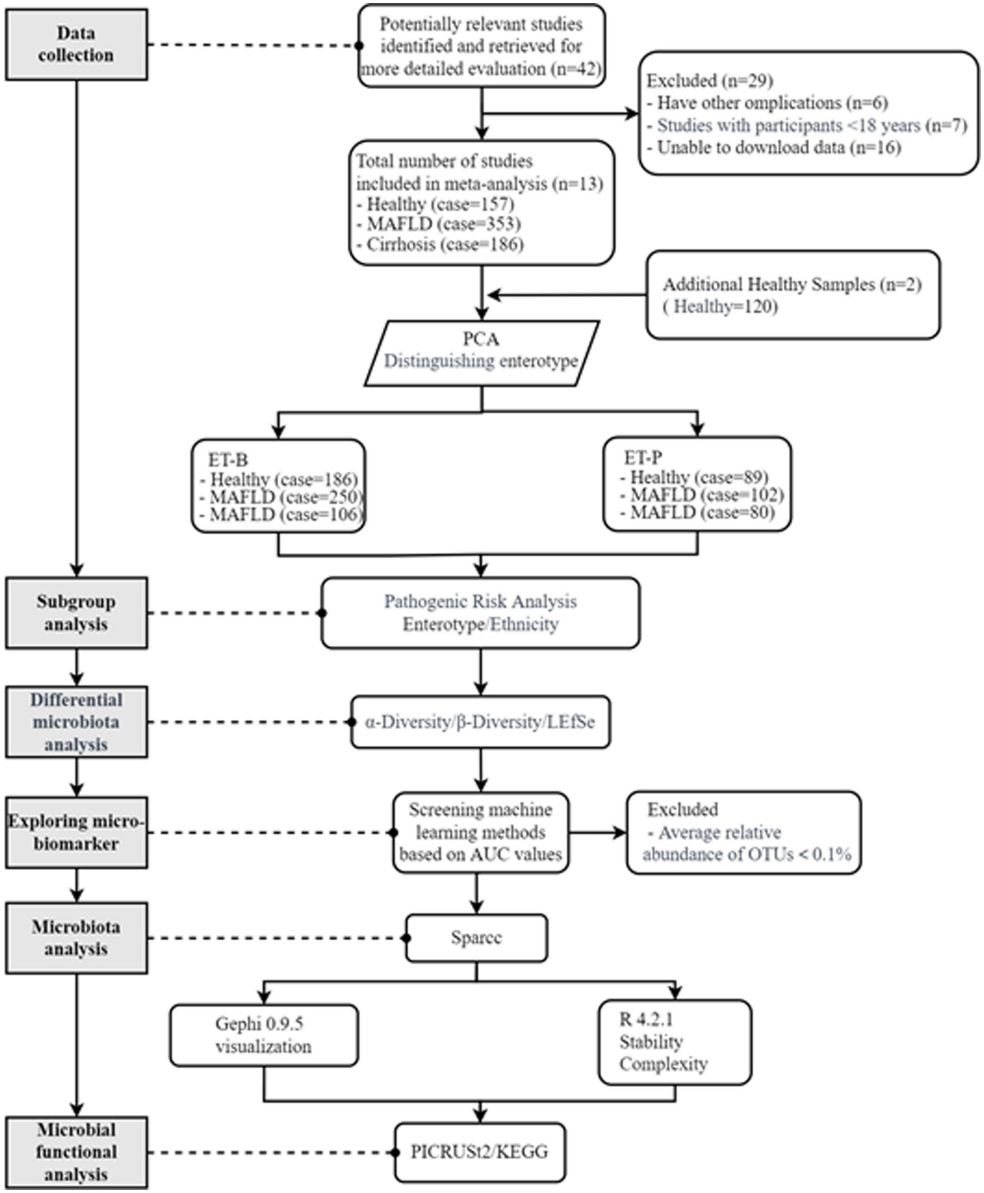
Flowchart of data selection and analysis for metabolic dysfunction-associated fatty liver disease (MASLD) and cirrhosis. ET-B, Bacteroides-dominated enterotype, ET-P, Prevotella-dominated enterotype. LEfSe, linear discriminant analysis effect size; OTUs, operational taxonomic units; Sparcc, Spearman correlation coefficients; KEGG, Kyoto Encyclopedia of Genes and Genomes.

The screening process is detailed in Figure 1. The studies were selected based on the new definition of fatty liver disease and met the diagnostic criteria for MASLD and liver cirrhosis in American Association for the Study of Liver Disease and European Association for the Study of the Liver. In these studies, MASLD was diagnosed using imaging or biopsy techniques, liver cirrhosis was diagnosed based on fibrosis scores calculated through blood tests, and liver stiffness was assessed by elastography or biopsy. A total of 42 studies were initially identified through preliminary screening, 29 of which were excluded for the following reasons: 6 studies involved other disease complications, 7 studies had participants under the age of 18, and 16 studies did not have gut microbiome data. Finally, 13 studies were included in the analysis (Study IDs: PRJNA246121, PRJNA382861, PRJNA541489, PRJNA518731, PRJNA559052, PRJEB40538, PRJNA860335, PRJNA431746, PRJNA445763, PRJNA449353, PRJNA471972, PRJNA748675, PRJNA967488), which involved 158 healthy volunteers, 353 MASLD patients, and 186 cirrhosis patients. Subgroup analysis and enterotype-based clustering were used to reduce inter-cohort variability and assess microbial community structure independent of technical batch effects. Due to the small sample size of healthy participants from studies from Australia and Italy, two additional analyses (Study IDs: PRJEB41867, PRJNA736583) were included, contributing 120 healthy volunteers. The basic characteristics of the participants from the studies included in the analysis are summarized in Supplementary Table 1.

### Subgroup analysis

**Figure 2:**
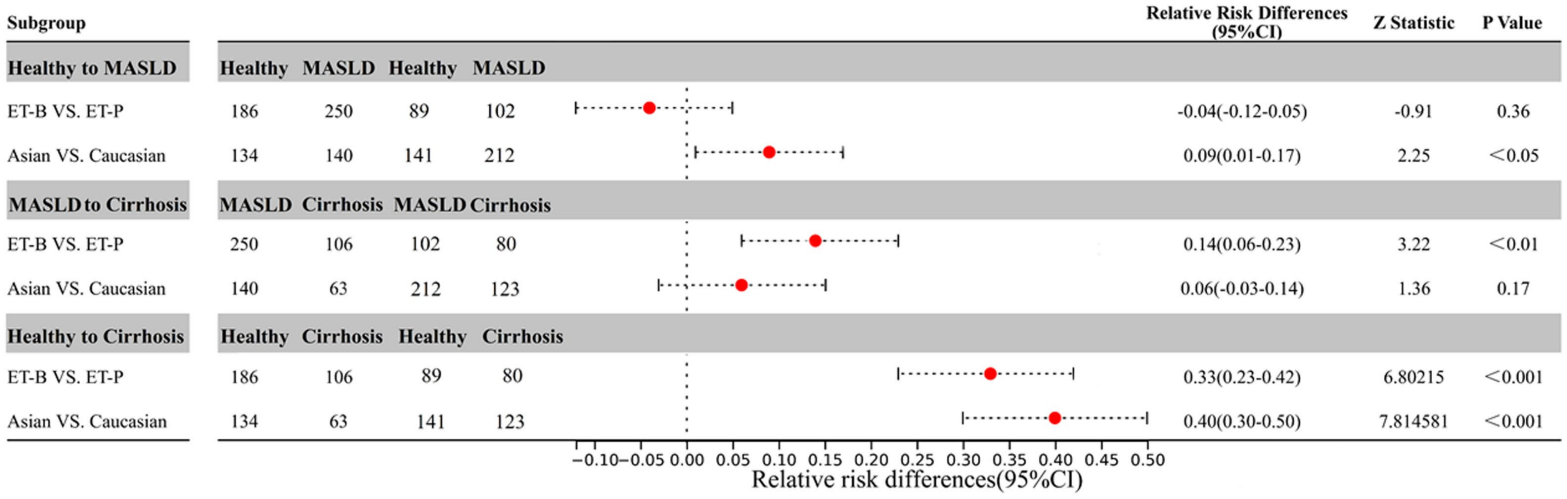
Forest plot of relative risk differences for MASLD and cirrhosis according to enterotypes and ethnicity. CI, confidence interval; MASLD, metabolic dysfunction-associated fatty liver disease; ET-B, Bacteroides-dominated enterotype, ET-P, Prevotella-dominated enterotype.

After data cleaning, a total of 813 data points were annotated with species information. Participants were then categorized into two gut microbiome clusters, ET-B and ET-P, based on the optimal clustering numbers (Supplementary Figures S1A,1B). In the enterotype analysis, principal component (PC) 1 and PC2 accounted for 47.38 and 21.17% of the gut microbiota variance respectively, representing a cumulative 68.55% of total variance. The model demonstrated statistically significant associations for specific variables and inter-group differences (*p* = 0.001). Cluster quality assessment using the CH index revealed optimal separation at k = 2 clusters, achieving a CH index of 242 that substantially exceeded values observed for other cluster numbers (Supplementary Figure S1A). This pattern indicated well-defined separation between enterotypes with high within-cluster consistency. Based on the gut microbiome samples, the 275 healthy participants were grouped into 186 in the ET-B and 89 in ET-P. The 352 MASLD participants included 250 in ET-B and 102 in the ET-P. The 186 cirrhosis participants included 106 in ET-B and 80 in ET-P (Figure 2).

As shown in Figure 2, there was no significant difference in the relative risk of MASLD between ET-B and ET-P when compared to the healthy group (*p* = 0.36). However, the relative risk of cirrhosis in ET-P was significantly higher than in ET-B when compared to the MASLD group (*p* < 0.05). Specifically, the relative risk difference between ET-B and ET-P was higher by 14% in the MASLD group compared to the cirrhosis group and by 33% among the healthy group compared to the cirrhosis group. Additionally, when ethnic differences were compared, Caucasians had a 9 and 40% higher relative risk of MASLD and cirrhosis, respectively, than Asians compared to the healthy group, although this difference did not reach statistical significance (*p* = 0.17). There was no significant difference in the relative risk of cirrhosis between Caucasians and Asians when compared to the MASLD group.

### *α*-diversity and primary bacteria in the healthy, MASLD, and cirrhosis groups according to enterotypes

**Figure 3:**
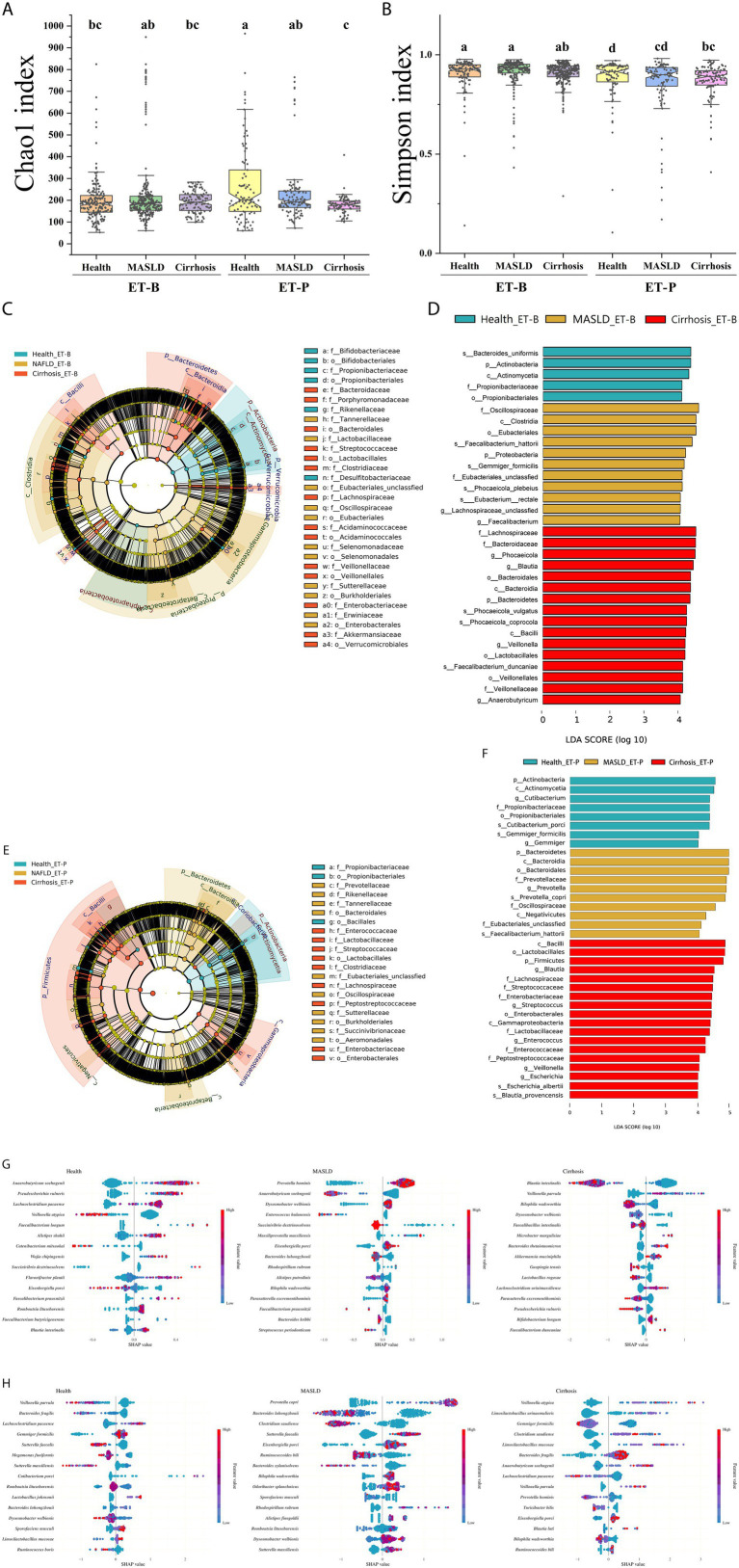
*α*-diversity and key microbial analysis of the gut microbiome. **(A,B)**. α-diversity measured by the Chao1 index and the Simpson index. **(C,D)** Evolutionary branching diagram and biomarker bar chart of ET-B’s Linear discriminant analysis effect size (LEfSe). **(E,F)** Evolutionary branching diagram and biomarker bar chart of ET-P’s Linear discriminant analysis effect size (LEfSe). The diagram shows taxonomic changes in the gut microbiome from the phylum to family levels. Red highlights taxa with higher relative abundance in the cirrhosis group, yellow indicates enrichment in the metabolic dysfunction-associated fatty liver disease (MASLD) group, and green represents taxa predominant in the healthy group. Only taxa with a linear discriminant analysis (LDA) score (Log 10) > 4 are displayed. **(G,H)** SHAP (SHapley Additive exPlanations) analysis of the ET-B and ET-P. ET-B, Bacteroides-dominated enterotype, ET-P, Prevotella-dominated enterotype.

The Chao1 index represents α-diversity based on observed rare species and estimated potential richness, independent of abundance distribution, while Simpson’s diversity index represents α-diversity, with a greater dependence on the weight of abundance distribution. As shown in Figures 3A,B, with respect to ET-B, there were no significant differences in the Chao1 and Simpson indices among the healthy, MASLD, and cirrhosis groups. However, with respect to ET-P, the Chao1 and Simpson indices were higher in the healthy participants than in the participants with cirrhosis (*p* < 0.05), indicating a loss of microbial richness and evenness, reflecting the ecological imbalance associated with cirrhosis in ET-P.

LEfSe was used to compare the microbiome features across the healthy, MASLD, and cirrhosis status in the two enterotypes, and the results are shown in Figures 3C–F. The microbiome taxa showed consistent trends of relative abundance across both enterotypes: Actinobacteria, Bacilli, and Betaproteobacteria were high in the healthy, cirrhosis, and MASLD groups, respectively. Inconsistencies in the two enterotypes included the following: The relative abundance of Bacteroidetes was higher in ET-B associated with cirrhosis and ET-P associated with MASLD; the relative abundance of Gammaproteobacteria was higher in ET-B associated with MASLD and ET-P associated with cirrhosis. Additionally, in ET-B associated with MASLD, the relative abundance of Proteobacteria and Clostridia was higher than the other bacteria in the group. In the ET-B among the healthy group, the relative abundance of Alphaproteobacteria was higher than other bacteria (Figures 3C,D). In ET-P associated with cirrhosis, Firmicutes had a higher relative abundance than other bacteria in the group (Figures 3E,F).

XGBoost was used to identify the stage-specific marker microbes at the species level as shown in Figures 3G,H. In ET-B, the relative abundance of *Anaerobutyricum soehngenii*, *Pseudescherichia vulneris*, and *Lachnoclostridium pacaense* was positively correlated with the healthy group, while *Prevotella hominis* and *Dysosmobacter welbionis* were positively correlated and *Anaerobutyricum soehngenii* negatively correlated with MASLD. Cirrhosis was positively correlated with *Veillonella parvula*, and negatively correlated with *Blautia intestinalis* and *Bilophila wadsworthia* (Figure 3G). In ET-P, the healthy group was positively correlated with *Lachnoclostridium pacaense* and negatively correlated with *Veillonella parvula* and *Bacteroides fragilis* (Figure 3H). *Prevotella copri* was positively correlated, while *Bacteroides luhongzhouii* and *Clostridium saudiense* were negatively correlated with MASLD. *Veillonella atypica* and *Limosilactobacillus urinaemulieris* had increased relative abundance, while the abundance of *Gemmiger formicilis* decreased in the cirrhosis group.

### *β*-diversity and core bacteria in the healthy, MASLD, and cirrhosis groups according to enterotypes

**Figure 4:**
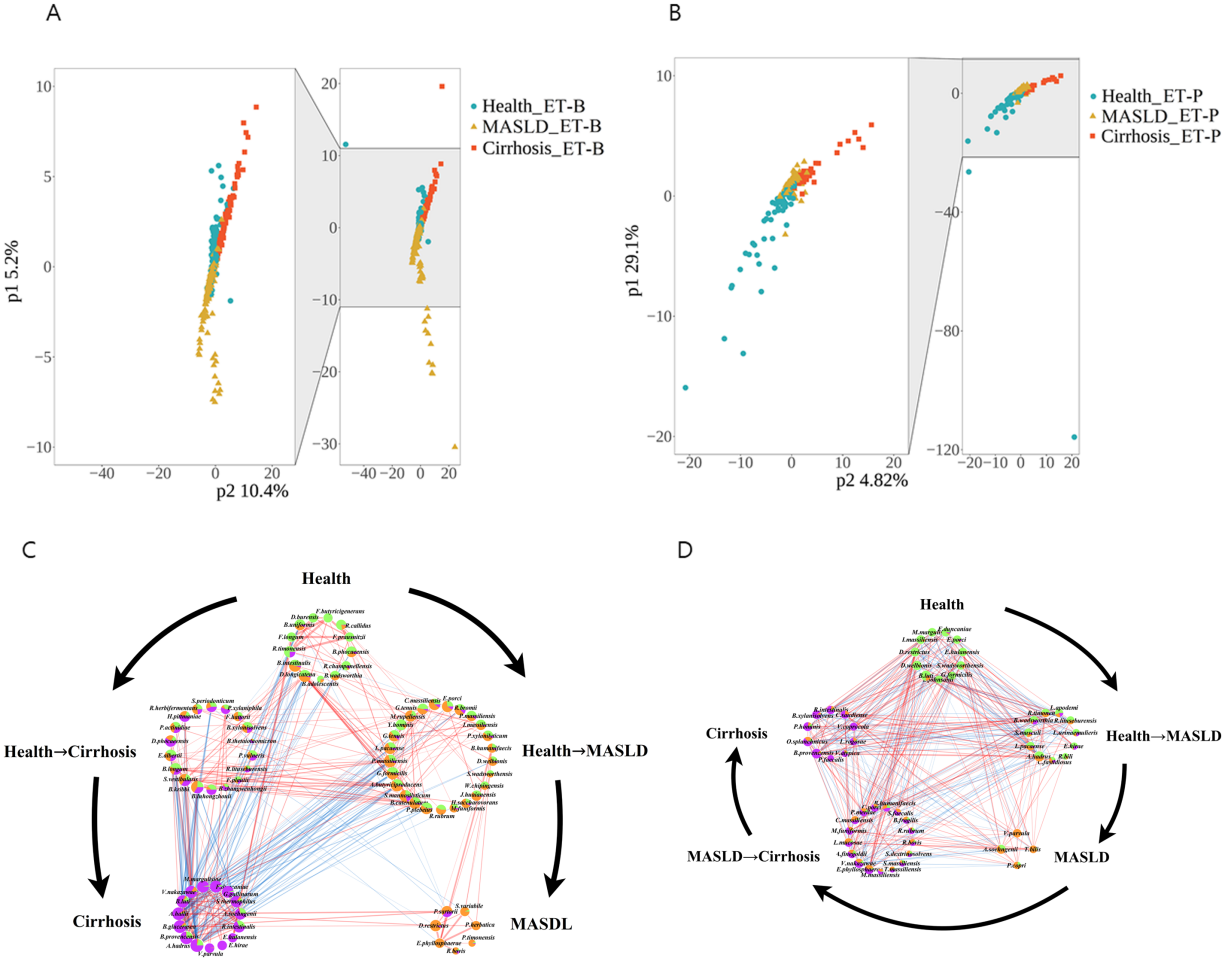
*β*-diversity analysis and gut microbiome interaction networks of metabolic dysfunction-associated fatty liver disease (MASLD) and cirrhosis gut microbiome determined according to ET-B and ET-P. **(A,B)** β-diversity in ET-B and ET-P determined by partial least squares discriminant analysis (PLS-DA). **(C,D)** Network analysis of bacteria in ET-B and ET-P. Interaction networks of the gut microbiome. Node size indicates degree, while sector proportions represent the relative abundance in each group. Red, yellow, and green colors correspond to the cirrhosis, healthy, and metabolic dysfunction-associated fatty liver disease (MASLD) groups, respectively. ET-B, Bacteroides-dominated enterotype, ET-P, Prevotella-dominated enterotype.

The β-diversity of the gut microbiome determined by PLS-DA is represented according to ET-B and ET-P in Figures 4A,B, respectively. Blue circles, yellow triangles, and red squares represent the healthy, MASLD, and cirrhosis samples, respectively. As observed in the figures, in ET-B, MASLD and cirrhosis were located on opposite sides of the healthy group, indicating that the microbial changes associated with MASLD and cirrhosis were distinct, suggestive of a trend diverging from healthy. On the other hand, in ET-P, the healthy, MASLD, and cirrhosis samples were arranged in a sequential pattern, suggesting that the microbial changes aligned with a continuous progression from healthy to MASLD to cirrhosis.

Furthermore, bacteria with similar relative abundance between the two disease states were categorized as part of an intermediate state. The intermediate states of the gut microbiome were defined as healthy−MASLD and healthy−cirrhosis in ET-B (Figure 4C) and as healthy−MASLD and MASLD−cirrhosis in ET-P (Figure 4D). The size and color of the circles represent the average relative abundance in different liver disease states. Green, yellow, and purple colors indicate the healthy, MASLD, and cirrhosis states, respectively. The overall gut microbiome of ET-B has a significant inhibitory effect on the cirrhosis-associated microbial community in the intermediate state of healthy-MASLD. However, the bacteria in the intermediates of healthy-MASLD included the ET-P microbiome associated with both MASLD and cirrhosis, indicating that the microbes in the intermediate states were more balanced in MASLD and cirrhosis.

The core microbiome in the healthy ET-B group primarily comprised Firmicutes (*Faecalibacterium prausnitzii*, *Faecalibacterium longum*), *Ruminococcus* (*Ruminococcus callidus*, *Ruminococcus champanellensis*), and *Blautia* (*Blautia intestinalis*, *Blautia phocaeensis*), as well as Actinobacteria (*Bifidobacterium adolescentis*) and Bacteroidetes (*Bacteroides uniformis*) (Figure 4C). In the healthy−MASLD stage of the ET-B, the core microbiome included Clostridiales (*Ruminococcus bromii*, *Intestinimonas massiliensis*, *Pseudoruminococcus massiliensis*, *Agathobaculum butyriciproducens*, *Lachnoclostridium pacaense*), Actinobacteria (*Bifidobacterium catenulatum*), and Bacteroidales (*Bacteroides humanifaecis*, *Phocaeicola plebeius*), with additional contributions from Burkholderiales (*Sutterella wadsworthensis*) and Rhodospirillales (*Rhodospirillum rubrum*) (Figure 4C). In the MASLD stage of the ET-B, the core microbiome comprised Clostridiales (*Ruminococcus bovis*, *Subdoligranulum* var*iabile*) and Bacteroidales (*Prevotella herbatica*, *Prevotellamassilia timonensis*, *Phocaeicola sartorii*) (Figure 4C). In the healthy−cirrhosis stage of the ET-B, the core microbiota included Firmicutes (*Faecalibacterium hattorii*, *Faecalibacterium prausnitzii*, *Faecalibacterium longum*, *Megasphaera elsdenii*), Actinobacteria (*Bifidobacterium bifidum*), Bacteroidetes (*Bacteroides caccae*), and Pseudomonadales (*Pseudomonas citronellolis*) (Figure 4C). In cirrhosis of the ET-B, the core microbiota was enriched in *Veillonella parvula*, and *Limosilactobacillus urinaemulieris*, with a high abundance of Proteobacteria and Firmicutes (*Ruminococcus* spp.) (Figure 4C).

The core microbiota of the healthy group in the ET-P gut type is primarily made up of Firmicutes, including Lachnospiraceae (*Blautia luti*, Dysosmobacter welbionis, *Gemmiger formicilis*), Ruminococcaceae (*Intestinimonas massiliensis* and *Faecalibacterium duncaniae*), and Lactobacillales (*Lactobacillus johnsonii* and *Enterococcus hulanensis*). The core microbiota during the healthy & MASLD stage includes Lachnospiraceae (*Anaerostipes hadrus* and *Lachnoclostridium pacaense*), Romboutsiaceae (Romboutsia timonensis and *Romboutsia lituseburensis*), and Lactobacillales (*Ligilactobacillus apodemi*, *Limosilactobacillus urinaemulieris*, and *Enterococcus hirae*). The core microbiota in the MASLD stage consists of Firmicutes (*Anaerobutyricum soehngenii*, *Veillonella parvula*, and *Turicibacter bilis*) and Bacteroidota (*Prevotella copri*). The MASLD & cirrhosis stage is characterized by Clostridiales (*Ruminococcus bovis*, *Catonella massiliensis*, *Tidjanibacter massiliensis*), Lactobacillales (*Limosilactobacillus mucosae*), Bacteroidales (*Bacteroides fragilis*, *Bacteroides humanifaecis*, *Parabacteroides merdae*, *Alistipes finegoldii*, and *Massiliprevotella massiliensis*), and some Proteobacteria (*Sutterella faecalis*, *Sutterella massiliensis*, *Erwinia phyllosphaerae*, and *Succinivibrio dextrinosolvens*). The cirrhosis stage is constituted by Clostridia (*Blautia provencensis*, *Roseburia intestinalis*, *Clostridium saudiense*, *Veillonella atypica*, *Lactobacillus rogosae*), Bacteroidales (*Odoribacter splanchnicus*, *Prevotella hominis*, *Paraprevotella xylaniphila*, and *Phocaeicola faecalis*), and Aeromonadales (*Vescimonas coprocola*).

### Toxicity scores of the core gut microbiota in liver disease states according to enterotypes

**Figure 5:**
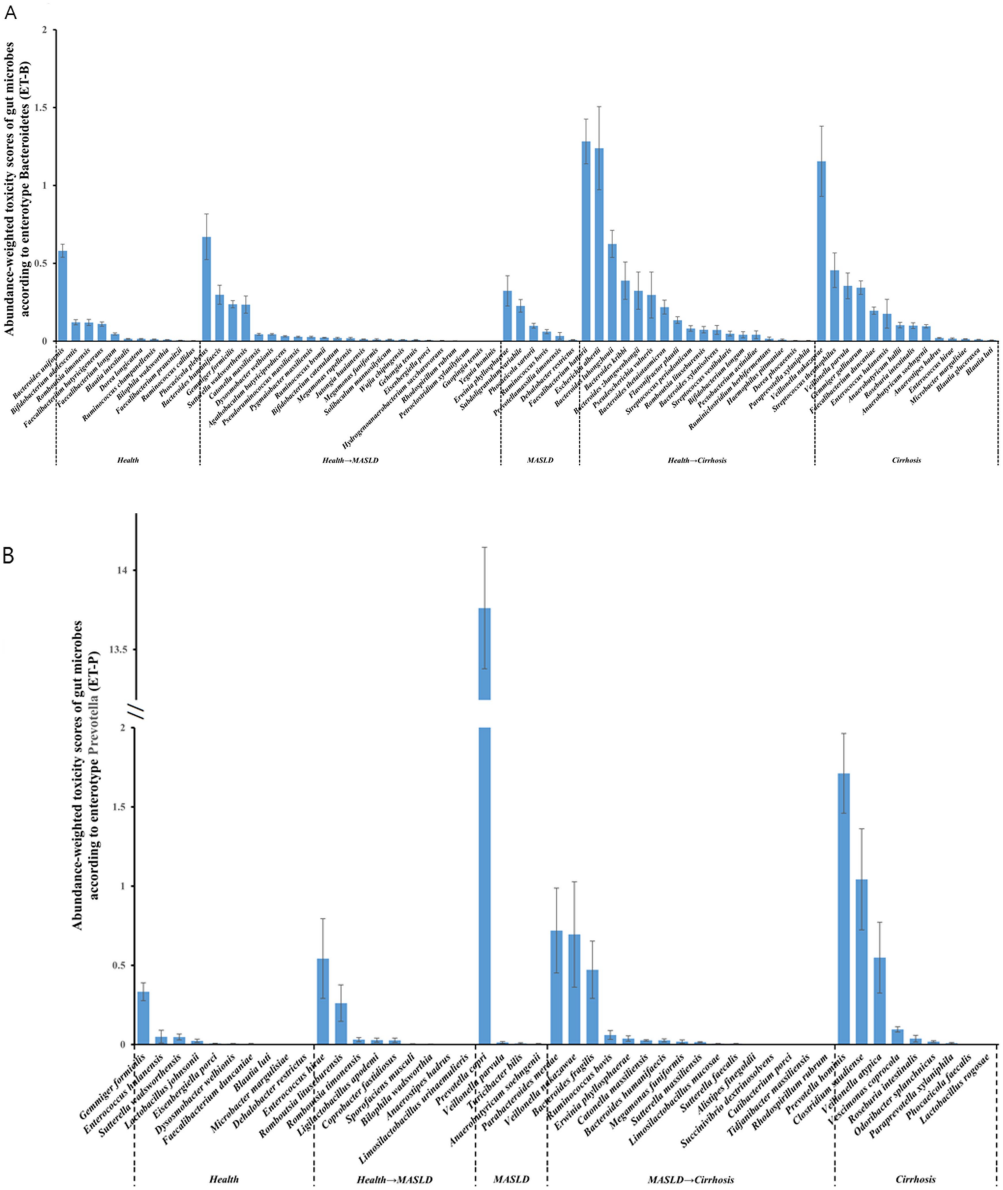
Toxicity analysis of gut microbiome. **(A,B)** Relative abundance-corrected toxicity scores of gut microbiota in ET-B and ET-P ET-B, Bacteroides-dominated enterotype, ET-P, Prevotella-dominated enterotype.

In ET-B associated with MASLD, fewer toxic microbes were present in the gut microbiome compared to the healthy group. However, during the cirrhosis stage, toxin levels increased significantly (Figure 5A). Specifically, *Faecalibacterium hattorii* (toxicity score: 1.28 ± 0.14) and *Escherichia albertii* (toxicity score: 1.24 ± 0.27) were positively associated with the progression of cirrhosis, while *Veillonella nakazawae* (toxicity score: 1.15 ± 0.23) continued to exacerbate the cirrhosis (Figure 5A). In contrast, the significant toxic bacterium in ET-P associated with MASLD was *Prevotella copri* (toxicity score: 13.76 ± 0.38), while the core toxic bacteria in cirrhosis were *Prevotella hominis* (toxicity score: 1.71 ± 0.25) and *Clostridium saudiense* (toxicity score: 1.04 ± 0.32) (Figure 5B).

### Metagenome function of gut bacteria by PICRUSt2 analysis

**Figure 6:**
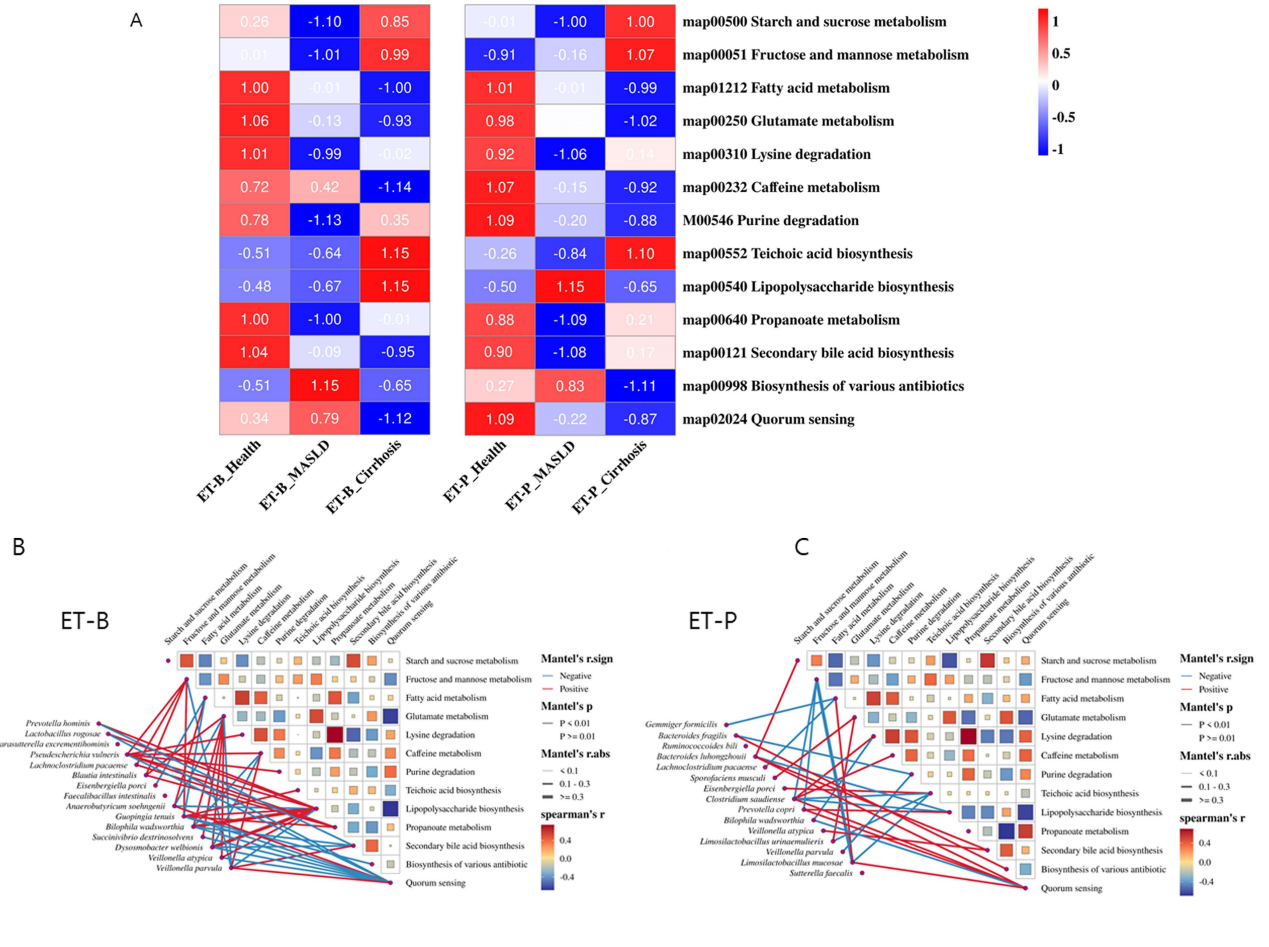
Functional analysis of gut microbiota. **(A)** Differences in nutrient metabolism. Data were standardized using Z-scores. **(B,C)** Network analysis of the correlation between gut microbiota and functions of ET-B and ET-P.

Figure 6A presents the predicted gut microbiome functions at different stages of the disease for the two gut types using PICRUSt2. The trends of the metagenome function of the gut bacteria across liver disease stages were similar in ET-B and ET-P. The ability to metabolize carbohydrates (map00500 and 00051) was positively associated with cirrhosis but inversely associated with MASLD in both enterotypes. Activity in the metabolism of fatty acids (map01212), proteins (map00310 and map00250), caffeine (map00232), purines (map00546), and short-chain fatty acid (SCFA)-related pathways (map000640) was inversely associated with MASLD and cirrhosis (Figure 6A). Additionally, endogenous antibiotics in the MASLD group (map00998) were positively associated, but were inversely associated with cirrhosis in both enterotypes. In contrast, some differences in metagenome function across the liver disease states between the enterotypes were observed: lipopolysaccharide (LPS) biosynthesis was positively associated with the cirrhosis stage in the ET-B and with the MASLD stage in the ET-P. Furthermore, the biosynthesis of secondary bile acids was significantly negatively correlated with the cirrhosis stage in the ET-B and with the MASLD stage in the ET-P (Figure 6A).

Figures 6B,C depict the interactions between differential bacterial species and metagenome functions. The thickness of the lines represents the strength of the interaction, with blue and red lines indicating inhibition and activation of the function by the microbe, respectively. The color and size of the rectangles represent the relationships between the microbiome functions with the same as those of the lines. We defined microbes with three or more significant function connections (edges) as key functional microbes. In the ET-B microbiome, *Parasutterella excrementihominis*, *Blautia intestinalis*, *Anaerobutyricum soehngenii*, *Guopingia tenuis*, *Dysosmobacter welbionis*, *Veillonella atypica*, and *Veillonella parvula* were dominant in metagenome functions (edges ≥ 3) (Figure 6B). Notably, *Anaerobutyricum soehngenii* was positively correlated with fatty acid metabolism in the gut microbiome and negatively correlated with LPS biosynthesis in the ET-B. *Prevotella hominis*, *Bilophila wadsworthia*, *Dysosmobacter welbionis*, and *Veillonella parvula* jointly regulated bile acid metabolism in the ET-B (Figure 6B).

In the ET-P, *Bacteroides luhongzhouii*, *Clostridium saudiense*, *Prevotella copri*, and *Limosilactobacillus mucosae* collectively influenced the gut microbiota’s nutrient metabolism and the biosynthesis of metabolic products (Figure 6C). *Gemmiger formicilis*, *Lachnoclostridium pacaense*, and *Limosilactobacillus mucosae* were negatively linked to fatty acid metabolism, while *Prevotella copri*, *Bacteroides luhongzhouii*, and *Clostridium saudiense* were positively associated with LPS biosynthesis. Furthermore, *Veillonella atypica* and *Bacteroides luhongzhouii* were positively associated with bile acid metabolism (Figure 6C).

## Discussion

This study demonstrated that the incidence of cirrhosis in ET-P was 33% higher than in ET-B, and this increase was significant. This was potentially linked to the fact that the microbiota in ET-P associated with MASLD promoted the progression to cirrhosis, while the core microbiota in ET-B associated with cirrhosis was largely suppressed by other gut microbes. Furthermore, the study identified specific gut microorganisms associated with cirrhosis: *Escherichia albertii* and *Veillonella nakazawae* were linked to cirrhosis in ET-B, while *Prevotella copri* was associated with MASLD, and *Prevotella hominis* and *Clostridium saudiense* were linked to cirrhosis in ET-P. In the gut metagenome function of both MASLD and cirrhosis, fatty acid and protein metabolism, as well as SCFA biosynthesis capacity, were reduced. Meanwhile, LPS biosynthesis increased, and secondary bile acid metabolism was abnormal in both MASLD and cirrhosis.

Numerous studies have highlighted significant differences in the incidence of liver diseases among different populations. These disparities have been attributed to lifestyles, regional economic conditions, and age (Herren et al., 2022). This study was novel, as it suggested that these differences could be related to the gut microbiota, with the incidence varying depending on the gut microbiome type. ET-P is primarily characterized by a Prevotella-dominant microbiome, with genus *Prevotella* accounting for an average of 33.9%, wherein *Prevotella copri* and *Prevotella hominis* are considered pathogenic strains linked to liver diseases, especially MASLD and cirrhosis. *Prevotella* are widely distributed in the oral cavity, gastrointestinal tract, and reproductive tract and are an important component of the gut microbiome. They ferment complex carbohydrates, such as cellulose, pectin, and polysaccharides. However, increasing evidence suggests that the excessive proliferation of *Prevotella* is associated with chronic inflammatory diseases and liver fibrosis (Sharma et al., 2022). Studies have also shown that *Prevotella*’s protoporphyrin IX and/or protoheme may contribute to liver injury, and endogenous hydrogen sulfide can increase serum interleukin (IL)-6 levels in patients (Gaddam et al., 2017). Notably, *Prevotella copri* has been associated with obesity, increased fasting blood glucose, and elevated insulin levels (Gong et al., 2024). Its mechanism appears to involve LPS biosynthesis and the overactivation of the immune system (De Filippis et al., 2019). Meanwhile, *Clostridium saudiense* has been strongly linked to diabetes risk (Kwan et al., 2022), with its mechanism involving secondary bile acids (Park et al., 2023a).

In contrast, ET-B, characterized by a Bacteroides-dominant gut microbiome, with *Bacteroides* accounting for 31.2%, showed that *Escherichia albertii* was associated with MASLD, while *Veillonella nakazawae* was linked to liver cirrhosis based on the toxin scores. *Escherichia albertii* is the second most pathogenic species in the *Escherichia* genus after *E. coli* and is considered an emerging intestinal pathogen in humans and animals (Muchaamba et al., 2022). The only reported toxins in *E. albertii* strains are cytolethal distending toxin (CDT) and Shiga toxin (Stx) (Hinenoya et al., 2017). Studies have shown that *E. albertii* can invade HeLa, Caco-2, and T84 cells, reducing epithelial resistance by redistributing tight junction proteins such as claudin-1 and zonula occludens-1, thereby increasing cell permeability (Yamamoto et al., 2017). Additionally, *Veillonella* metabolizes lactate and releases acetate, which triggers gluconeogenesis and lipogenesis, leading to increased lipid storage in the liver and body tissues (Zhang and Huang, 2023). *Veillonella* has also been shown to play a role in small intestinal bacterial overgrowth (SIBO) (Zhan et al., 2022), a condition more common in overweight and obese individuals (Palmas et al., 2021). SIBO is associated with the increased expression of toll-like receptor-4 (TLR4) and secretion of IL-8, which influence the inflammatory pathways involved in the pathogenesis of MASLD and cirrhosis (Ghosh and Jesudian, 2019).

An important finding of this study was that gut microbes promote liver inflammation through LPS in MASLD and cirrhosis. Interestingly, this study found significant differences between the two enterotypes, with increased LPS levels in the cirrhosis stage of ET-B and the MASLD stage of ET-P. LPS released from the dead gram-negative bacteria cell walls is recognized by dendritic cells or activated via Treg cells, triggering the adaptive immune system (Alpizar et al., 2017). This process activates TLRs, leading to nuclear factor-κB (NF-κB) signaling and the activation of NOD-like receptors (NLR) through pathogen-associated molecular patterns (PAMPs) (Cario, 2020). As a result, inflammatory cytokines and chemokines are transported through the mesenteric venous system to the portal vein and ultimately to the liver (Brandl et al., 2017). In the liver, the Kupffer cells become activated, and the associated inflammatory factors further damage the intestinal mucosa. Elevated endotoxins and pro-inflammatory cytokines stimulate the activation and proliferation of hepatic stellate cells, leading to the secretion of the extracellular matrix and the promotion of liver fibrosis, which facilitates the progression of cirrhosis (Lee et al., 2017). Additionally, the decreased phagocytic capacity of the Kupffer cells, combined with hemodynamic changes in cirrhosis, disrupts intestinal secretion, absorption, barrier function, and circulation, ultimately resulting in further gut barrier damage (Nesci et al., 2024). This also leads to metabolic disturbances. Cirrhotic patients exhibit significantly elevated tumor necrosis factor (TNF)-*α* secretion, which affects tight junction (TJ) integrity (Jiang X. S. et al., 2023). In the decompensated phase of cirrhosis, activated intestinal macrophages secrete TJ-modulating factors like nitric oxide (NO) and IL-6, contributing to the disruption of the intestinal epithelial barrier. This damage to the gut barrier further impairs the repair of the liver structure and function, creating a vicious cycle between the gut and the liver (Rodrigues et al., 2024). Therefore, gut microbiota might be differently involved in the mechanism of progression of MASLD and cirrhosis.

Another important finding of the present study was the abnormal increase in secondary bile acid biosynthesis in liver disease, specifically in ET-B during the cirrhosis stage and in ET-P during the MASLD stage. This differential pattern may be linked to the involvement of Bacteroides, which play a key role in secondary bile acid biosynthesis (Yu et al., 2021). Due to a vicious cycle between the liver and gut, some studies have found that secondary bile acids suppress the expression of membrane-bound chemokine (C-X-C motif) ligand 16 (CXCL16) in liver sinusoidal endothelial cells and reducing the number of natural killer T cells, which contributes to liver cancer development (Jia, 2019). In a mouse model of MASH, supplementation with deoxycholic acid (DCA) enhanced farnesoid X receptor (FXR) and Takeda G protein-coupled receptor 5 (TGR5) signaling, improving glucose tolerance and insulin resistance, and reducing liver steatosis (Oseini and Sanyal, 2017). Ursodeoxycholic acid (UDCA) has been shown to regulate the protein kinase B/mammalian target of rapamycin/sterol regulatory element-binding protein-1 (AKT/mTOR/SREBP-1) signaling pathway, reducing lipid accumulation in MASLD cell models induced by oleic acid (Chen et al., 2018). This may help decrease hepatic lipid accumulation, improve liver function, and prevent the progression and exacerbation of liver diseases.

Although this study implemented rigorous data management protocols and robust analytical techniques, several limitations must be acknowledged. First, dietary patterns—a known determinant of gut microbiota—could not be uniformly assessed across cohorts, representing a potential confounding factor. Geographic variability also constrains generalizability, as regional and ethnic differences influence microbiome composition. Medication usage, especially antibiotics and proton pump inhibitors, was not consistently recorded and may have impacted microbiota profiles. Second, while we employed 5-fold cross-validation within the XGBoost modeling framework to reduce overfitting and ensure model robustness, the absence of an independent external dataset limits the generalizability of our findings. Without external or cross-cohort validation, the predictive model’s performance across diverse populations remains uncertain and should be interpreted with caution. Third, the cross-sectional design precludes causal inference or temporal mapping of microbiota changes during disease progression. Although batch effects were addressed through uniform preprocessing and subgroup analysis, cohort-specific biases may persist. Fourth, MASLD and MASH stages were not distinctly analyzed, as most included studies grouped them under a single disease category, potentially obscuring stage-specific microbial signatures. Finally, our use of PICRUSt2 for functional prediction, though widely accepted, offers only inferred, not directly measured, functional profiles based on 16S rRNA data. Given the reliance on reference genomes, predictive limitations are especially relevant in disease contexts where novel or poorly characterized taxa may play significant roles. Thus, pathway inferences such as LPS biosynthesis and bile acid metabolism should be interpreted with caution. Future studies should integrate multi-omics approaches—such as metagenomics, metatranscriptomics, and metabolomics—to validate functional predictions with greater biological resolution in longitudinal designs.

In summary, our systematic investigation of gut microbiome characteristics in MASLD and cirrhosis provided critical insights into the intricate interactions between microbial communities and the progression of liver disease. The findings revealed that enterotype-specific microbial signatures substantially modulate the disease trajectory, demonstrating the critical role of gut microbiota in the pathogenesis of MASLD and cirrhosis. Key discoveries included significant variations in microbial composition across different enterotypes, identification of distinct bacterial strains associated with MASLD and cirrhosis progression, and metabolic functional analyses uncovering altered metabolic pathways in MASLD and cirrhosis. These findings offer promising opportunities for developing enterotype-specific diagnostic biomarkers and precision medicine approaches, potentially enabling early detection of disease, risk stratification, and targeted therapeutic interventions. Future research should prioritize validating the identified microbial markers in prospective clinical studies, elucidating the causal mechanisms linking specific bacterial strains to liver disease progression, developing microbiome-based personalized therapeutic strategies, and exploring probiotic or prebiotic interventions tailored to specific enterotypes.

## Data Availability

The original contributions presented in the study are included in the article/Supplementary material, further inquiries can be directed to the corresponding authors.

## Footnotes

^1^ https://www.prisma-statement.org/prisma-2020-statement
