# Supplementary material for: Enterotype-stratified gut microbial signatures in MASLD and cirrhosis based on integrated microbiome data
Source: Front Microbiol. 2025 May 15;16:1568672. doi: 10.3389/fmicb.2025.1568672 (PMC12121994; doi:10.3389/fmicb.2025.1568672)
Supplement: Supplementary file 1 [file Data_Sheet_1.pdf]

Supplementary materials

Supplementary figure S1. Participants were classified into two enterotypes based on the optimal number of clusters.

A

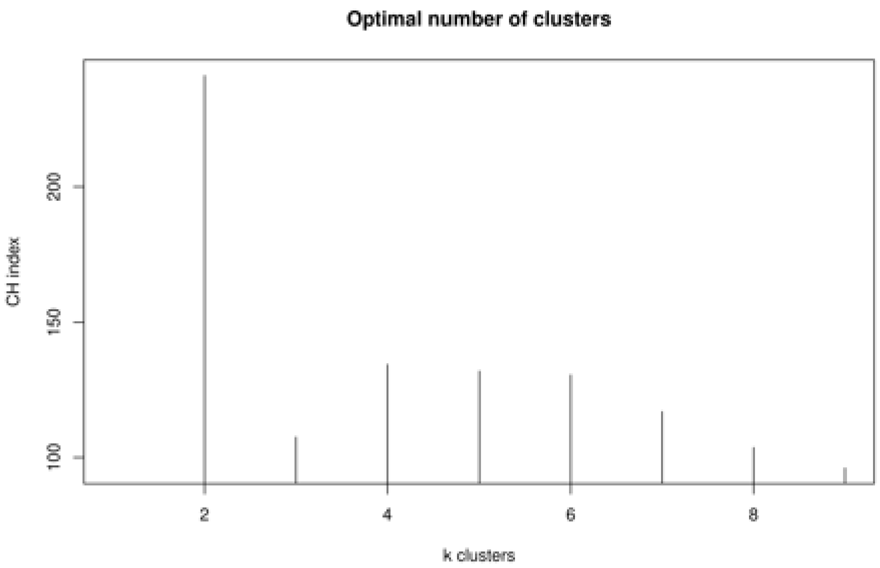

B

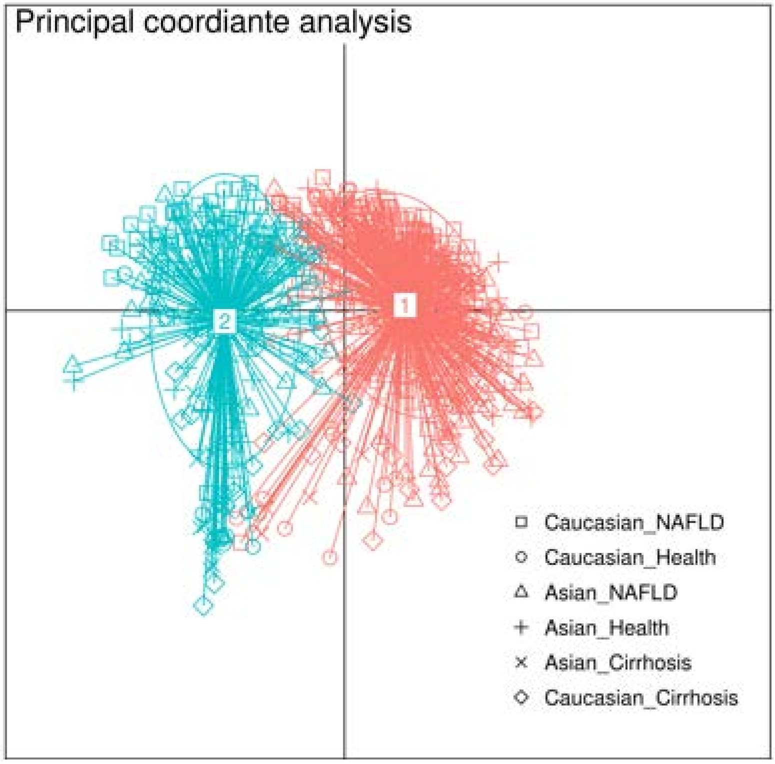

At the family level, 1 and 2 mainly consisted of Bacteroidaceae (ET-B) and Prevotellaceae (ET-P). At the genus level, they primarily included *Bacteroides* and *Prevotella*, respectively. ET-B, Bacteroides-dominated enterotype, ET-P, Prevotella-dominated enterotype

Table S1. The characteristics of each selected three-arm cohort from health and MASLD and cirrhosis.

Study(ID)	Health	NAFLD	CIR	Year	City, Country
PRJNA246121	32	53	0	2015	Beijing, China
PRJNA382861	25	31	0	2017	Shanghai, China
PRJNA541489	23	24	0	2019	Beijing, China
PRJNA518731	0	36	0	2020	Auckland,New Zealand
PRJNA559052	0	86	0	2020	Leuven, Belgium
PRJEB40538	0	91	0	2021	Italy; Greece; Serbia
PRJNA860335	29	32	0	2022	Beijing, China
PRJNA431746	8	0	88	2018	Styria, Austria
PRJNA445763	20	0	36	2018	Harbin, China
PRJNA449353	5	0	5	2019	Nanjing, China
PRJNA471972	14	0	35	2020	Rome, Italy
PRJNA748675	1	0	1	2022	Taoyuan, Taiwan
PRJNA967488	0	0	21	2023	Chengdu, China
PRJEB41867	100	0	0	2020	Graz, Austria
PRJNA736583	20	0	0	2022	Naples, Italy
**Asians**	**135**	**140**	**63**		
**Europeans**	**142**	**213**	**123**		
**Total**	**277**	**353**	**186**		
